# Dietary zinc enrichment reduces the cadmium burden of mealworm beetle (*Tenebrio molitor*) larvae

**DOI:** 10.1038/s41598-020-77079-x

**Published:** 2020-11-18

**Authors:** Claudia Keil, Maria Maares, Nina Kröncke, Rainer Benning, Hajo Haase

**Affiliations:** 1 Institute for Food Technology and Food Chemistry, Technische Universität Berlin, Straße des 17. Juni 135, 10623 Berlin, Germany; 2 Institute of Food Technology and Bioprocess Engineering, University of Applied Sciences Bremerhaven, An der Karlstadt 8, 27568 Bremerhaven, Germany

**Keywords:** Nutrition, Nutritional supplements, Toxicology

## Abstract

The industrial production of *Tenebrio molitor* L. requires optimized rearing and processing conditions to generate insect biomass with high nutritional value in large quantities. One of the problems arising from processing is a tremendous loss in mineral accessibility, affecting, amongst others, the essential trace element Zn. As a feasible strategy this study investigates Zn-enrichment of mealworms during rearing to meet the nutritional requirements for humans and animals. Following feeding ZnSO_4_-spiked wheat bran substrates late instar mealworm larvae were evaluated for essential micronutrients and human/animal toxic elements. In addition, growth rate and viability were assessed to select optimal conditions for future mass-rearing. Zn-feeding dose-dependently raised the total Zn content, yet the Zn_larvae_/Zn_wheat bran_ ratio decreased inversely related to its concentration, indicating an active Zn homeostasis within the mealworms. The Cu status remained stable, suggesting that, in contrast to mammals, the intestinal Cu absorption in mealworm larvae is not affected by Zn. Zn biofortification led to a moderate Fe and Mn reduction in mealworms, a problem that certainly can be overcome by Fe/Mn co-supplementation during rearing. Most importantly, Zn feeding massively reduced the levels of the human/animal toxicant Cd within the mealworm larvae, a technological novelty of outstanding importance to be implemented in the future production process to ensure the consumer safety of this edible insect species.

## Introduction

World population is projected to reach ten billion people by the middle of the century. The United Nations therefore assume that between 2050 and 2070 food production needs to be twice as high as today in order to meet the expected increase in consumption^1^. Consequently, alternative food sources are becoming increasingly important. According to the Food and Agriculture Organization of the United Nations, insects as an alternative environmentally friendly food and feed source are amongst the nutrients of choice for ensuring food security for the growing world population^2^. Just recently Europe has opened the utilization of insects for food (EU regulation 2015/2283^3^) and feed (EU 2017/893^4^). There is an urgent need to define globally harmonized quality standards for animal farming, processing and marketing in view of food safety, nutritional quality, and consumer demands^5,6^. In 2015, the EFSA published a scientific opinion on a risk profile related to the production and consumption of insects as food and feed^7,8^. Amongst the insects evaluated, the yellow mealworm (*Tenebrio molitor* L., Coleoptera: Tenebrionidae) has received extensive attention because of its high macronutrient value (on a dry weight basis $\sim$ 55% protein, 30% lipids, 7% carbohydrate)^9^ and its wide range of potential applications^10,11^. This makes these mealworms ideal candidates for cultivation and processing on an industrial scale^12^. With regard to economics and environmental issues, efforts have been made to improve mass production of the yellow mealworm by utilizing alternative raw materials with high nutritional value and low cost. In particular, wheat bran, a by‐product of industrial wheat flour milling amounting to about 150 million tons per year^13^, represents a highly economic, low-cost source of valuable nutrients for animals, including insects^10,14,15^. Recent studies showed the benefits of admixing agri-food industry by-products into wheat flour/bran for mealworm larvae rearing in terms of improved larval growth, diminished microbial load and improved antioxidant status^16,17^. Moreover, co-feeding of lipid- and protein-based supplements had a positive effect on macronutrient composition of mealworm larvae, upgrading them for food and feed applications^18^.

Little is known about the specific metabolic and nutritional response of yellow mealworms to alterations in dietary micronutrient supply. *T. molitor* larvae in principle are rich in vitamins and minerals^9^. The in vitro solubility and availability of essential minerals, particularly Zn, from fresh mealworms, however, is rather poor^19^. Mealworm drying even aggravates this problem^20^. A promising strategy for improving the mineral quality of *T. molitor* could be the use of Zn-enriched feed during breeding^20^. In this regard, the mealworm Zn tolerance needs to be determined, in order to minimize feed-aversion induced growth delays and mortality during rearing^21^. Moreover, excess Zn and minerals ingested with the feed may compete for intestinal transporters and intracellular chaperones in order to be efficiently delivered across the gut epithelium into the hemolymph. For mammals, intestinal competition between Zn and several other essential micronutrients (Cu, Fe, Ca) or toxic heavy metals (Pb, Cd) has been described^22,23^. Hence, the aim of this study was to investigate the potential dimensions of Zn enrichment in *T. molitor* during late instar larval development, when providing a ZnSO_4_-spiked wheat brain diet. Furthermore, it was to be investigated to what extent this fortification impacts the larval composition in essential and toxic elements, respectively. These results will be of utmost importance when aiming to improve *T. molitor* processing technologies, a basic prerequisite for utilizing mealworms as novel food or animal feed in the future.

## Results

### Larval growth parameters

**Figure 1:**
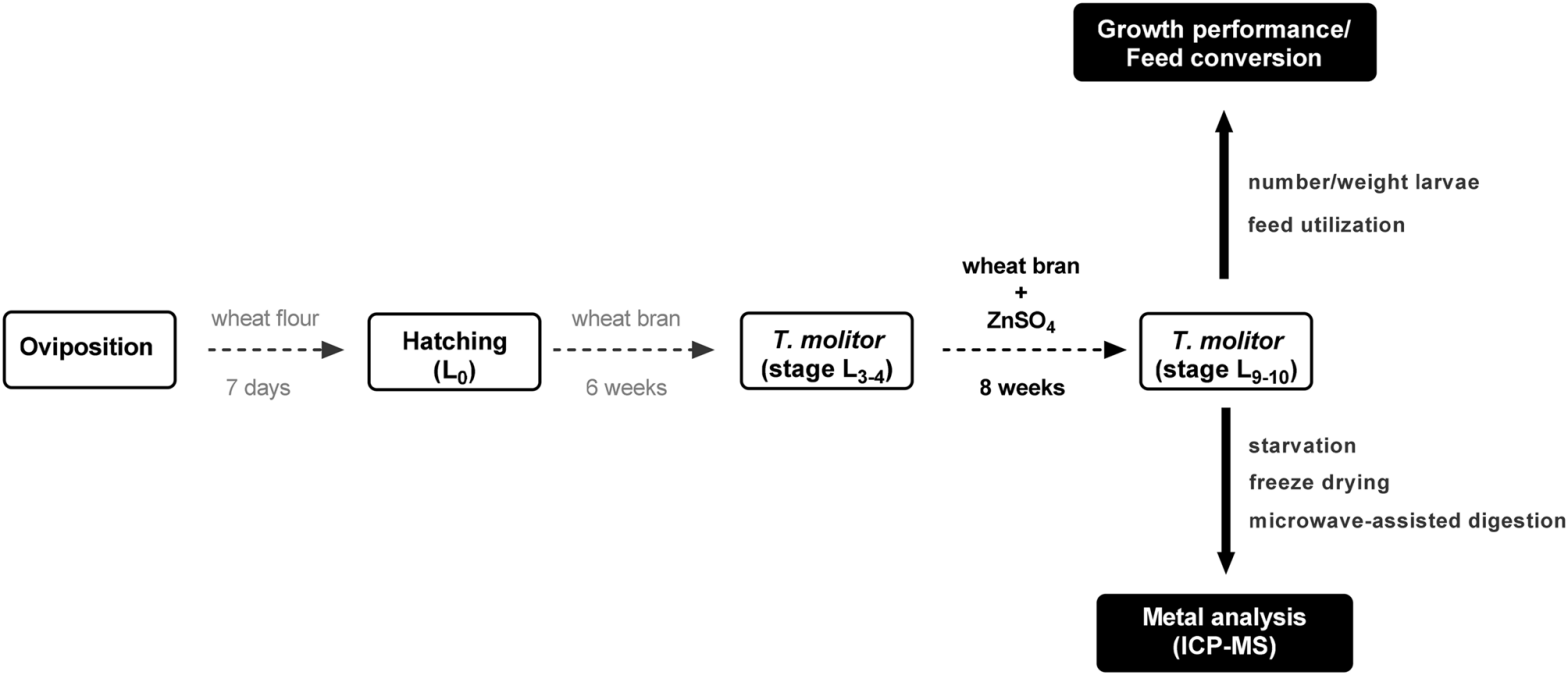
Procedure of the mealworm Zn-fortification experiment.

Diet composition is one of the main variables determining the efficiency of feed conversion into biomass for a given insect species in industrial mass rearing. *T. molitor* larvae fed over the whole period with Zn_basal_ wheat bran (Fig. 1) did effectively increase their starting L_3–4_ weight by a factor of 40, with very low mortality rate (Table 1). Overall 15.0 ± 0.5 g (around 75%) of the total offered Zn_basal_ wheat bran material was consumed (FC) with a total assimilation (FA) of 6.7 ± 0.3 g. The increase in larval body weight is reflected in a favorable feed conversion ratio (FCR 5.1 ± 0.1) and feed conversion efficiency (ECI 19.5 ± 0.3%) (Table 1). Mealworms grown in any of the Zn-enriched wheat bran materials also remained highly viable until late instar, but were significantly lower in fresh weight at stage L_9–10_ (52.3 ± 1.6 mg Zn_basal_ vs. 42.4 ± 1.8 mg Zn_40_; ANOVA with Dunnett’s post hoc test Zn_basal_ vs Zn_40_ p < 0.01), corresponding to a loss in biomass of around 20% (Table 1). Total feed consumption over the eight weeks feeding period was almost the same for the different groups, suggesting that the animals had no aversions against the spiked wheat bran, even though the added ZnSO_4_ was in the range of gustatory impact (0.35% w/w in the Zn_40_ wheat bran^21^). Likewise, total feces production was comparable, indicating that the overall amount of feed available for assimilation and growth (FA) was similar between the different feeding groups. Nevertheless, the slightly diminished feed conversion efficiency (ECI and ECD) of the Zn-enriched wheat bran (Table 1) suggests certain post-absorptive nutrient imbalances of the larvae, when starting from Zn-enriched bran, which seems to be restrictive for mealworm growth and development.

**Table 1:** Larval growth and feed utilization parameters.

Feeding group ^#^	LWG_total_ start L_3–4_ [g]	LWG_total_ end L_9–10_ [g]	Average weight larvae end L_9–10_ [mg]	Survival rate [%]	FC [g]	ECI [%]	FCR	Feces [g]	FA [g]	ECD [%]
Zn_basal_	0.07 ± 0.0	2.9 ± 0.1	52.3 ± 1.6	95.7 ± 1.1	15.0 ± 0.5	19.5 ± 0.3	5.1 ± 0.1	8.4 ± 0.3	6.7 ± 0.3	44.1 ± 1.0
Zn_2.5_	0.07 ± 0.0	3.0 ± 0.1	52.6 ± 0.7	97.0 ± 1.4	17.9 ± 0.3*	16.6 ± 0.2***	6.0 ± 0.1***	9.3 ± 0.3	8.6 ± 0.2**	34.5 ± 0.6***
Zn_5_	0.07 ± 0.0	2.8 ± 0.1	50.6 ± 1.0	96.0 ± 2.3	16.9 ± 0.5	16.7 ± 0.3***	6.0 ± 0.1***	8.7 ± 0.4	8.3 ± 0.1**	34.2 ± 0.7***
Zn_7.5_	0.07 ± 0.0	2.6 ± 0.1*	44.6 ± 2.5*	98.3 ± 0.7	15.9 ± 1.0	16.1 ± 0.2***	6.2 ± 0.1***	8.2 ± 0.7	7.7 ± 0.3	33.0 ± 0.6***
Zn_10_	0.07 ± 0.0	2.6 ± 0.1*	45.2 ± 0.8*	98.3 ± 0.5	15.5 ± 0.2	16.7 ± 0.6***	6.0 ± 0.2***	7.8 ± 0.4	7.7 ± 0.2	33.5 ± 0.2***
Zn_15_	0.07 ± 0.0	2.6 ± 0.1*	45. 2 ± 1.8*	97.0 ± 1.5	15.1 ± 0.5	16.9 ± 0.2***	5.9 ± 0.8***	7.7 ± 0.4	7.4 ± 0.1	34.7 ± 1.3***
Zn_20_	0.07 ± 0.0	2.4 ± 0.2**	42.3 ± 2.2**	97.7 ± 1.2	14.6 ± 0.9	16.5 ± 0.1***	6.0 ± 0.1***	7.4 ± 0.6	7.1 ± 0.4	33.7 ± 0.6***
Zn_25_	0.07 ± 0.0	2.4 ± 0.1***	41.6 ± 1.9***	98.7 ± 0.8	14.1 ± 0.4	16.9 ± 0.5***	5.9 ± 0.2***	6.9 ± 0.2	7.2 ± 0.1	33.3 ± 0.9***
Zn_30_	0.07 ± 0.0	2.5 ± 0.1*	43.9 ± 2.3*	99.3 ± 0.7	15.1 ± 0.8	16.8 ± 0.3***	5.9 ± 0.1***	7.8 ± 0.5	7.3 ± 0.3	34.6 ± 0.4***
Zn_40_	0.07 ± 0.0	2.4 ± 0.1**	42.4 ± 1.8**	97.7 ± 0.8	14.0 ± 0.4	17. 2 ± 0.4***	5.8 ± 0.1***	6.5 ± 0.5	7.4 ± 0.7	33.1 ± 2.0***

*T. molitor* larvae (start: 60 larvae; developmental stage L_3–4_) were fed with Zn-spiked wheat bran feed for 8 weeks (end: developmental stage L_9–10_); # see Table 2 for total Zn content of the wheat bran material. LWG_total_/ total live weight gain larvae; average larval weight; FC/total feed consumption; ECI/ efficiency of ingested feed conversion; FCR/ feed conversion ratio; total feces release during rearing; FA/ feed assimilation; ECD/ efficiency of digested feed conversion; Data are shown as means ± SEM of 5 replicates. Statistically significant differences from control are indicated (*p < 0.05, **p < 0.01, ***p < 0.001) one-way ANOVA with Dunnett’s post hoc test.

### Zn-enrichment in *T. molitor* larvae

**Figure 2:**
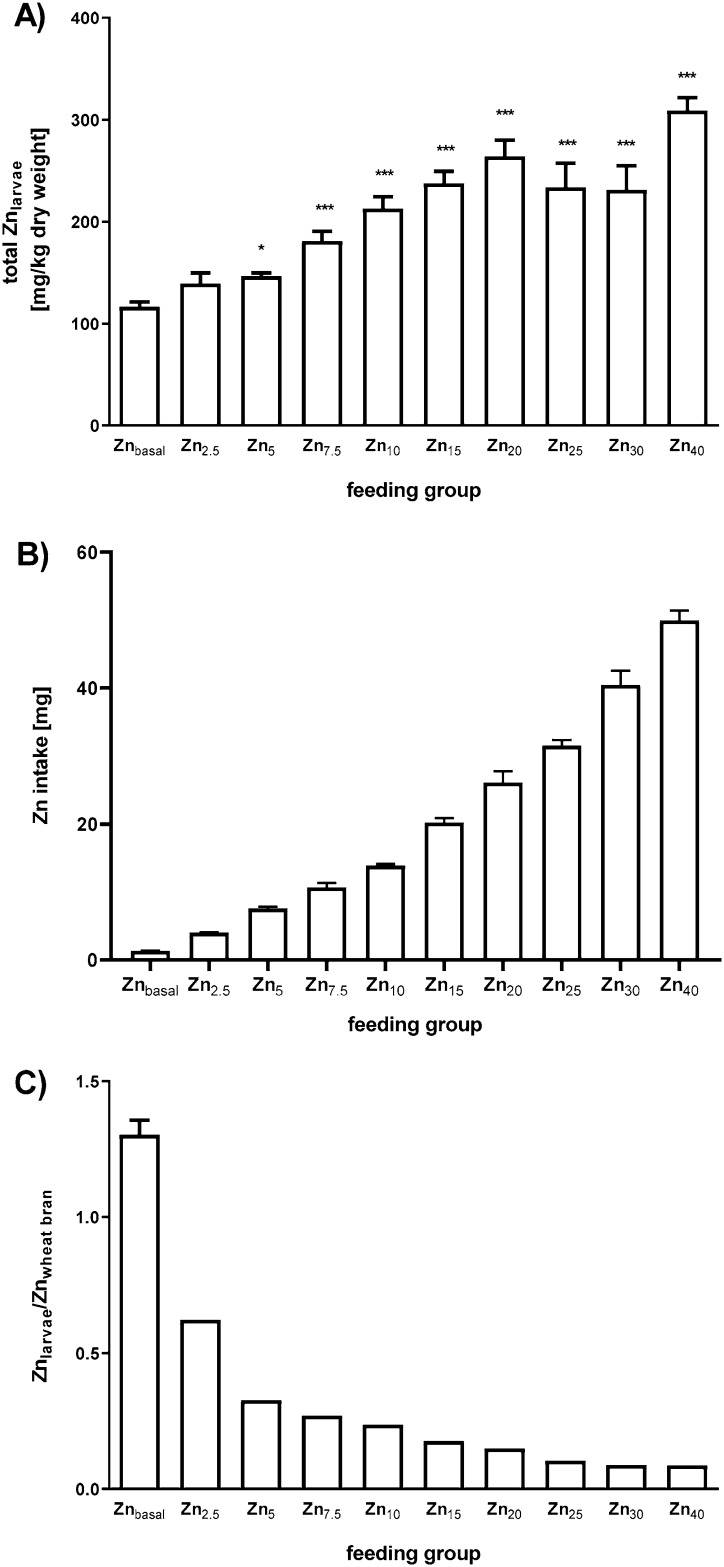
Zn concentrations in the Zn-biofortified *T. molitor* larvae. *T. molitor* larvae were provided for 8 weeks with either Zn_basal_ wheat bran or wheat bran material spiked with ZnSO_4_·7H_2_O up to fourfold of basal Zn content (see Table 1). (**A**) Larval Zn content determined by ICP-MS normalized to the weight of the freeze dried animals. Statistically significant differences from control are indicated (*p < 0.05, **p < 0.01, ***p < 0.001; one-way ANOVA with Dunnett’s post hoc test) (**B**) Total Zn intake over the feeding period was calculated from feed consumption data (see Table 2). (**C**) Zn_larvae_/Zn_wheat bran_ ratio. Data are shown as means + SEM of five replicates.

Based on FC, total Zn intake for the Zn_basal_ group during the feeding period was estimated to be 1.4 ± 0.1 mg ($\sim$ 24.3 ± 0.9 µg Zn/ animal; Fig. 2B). Total Zn content in the final biomass of the Zn_basal_ L_9–10_ larvae was 116.4 ± 4.3 mg kg^−1^ dry weight (Fig. 2A). Yet the larvae are higher in total Zn than the feeding material, indicating an accumulation of the essential mineral within the animals (Fig. 2C). Wheat bran contains significant amounts of phytate*,* an anti-nutrient inhibiting intestinal Zn absorption in both invertebrates and vertebrates^24,25^. Assuming a typical phytate content of $\sim$ 6000 mg/100 g wheat bran^26^, the estimated phytate/Zn molar ratio for the Zn_basal_ feed corresponds to 66, which might be disadvantageous for larval midgut Zn accessibility. In the Zn-spiked feed this ratio is shifted in favor of Zn, decreasing the molar phytate/Zn-ratio to 3.8 for the Zn_5_-wheat bran and down to 1.7 for Zn_40_-wheat bran. We observed a rise in larval Zn content with elevated level of Zn in the wheat bran, yielding up to a maximum of 309.0 ± 0.5 mg Zn kg^−1^ larval dry mass for the Zn_40_ group (Fig. 2A). The Zn_larvae_/Zn_wheat bran_ ratio, however, decreased with increasing Zn concentration in the feed from 1.3 ± 0.1 for the Zn_basal_ group down to 0.1 ± 0.0 for Zn_40_ feed larvae (Fig. 2C), suggesting an active regulation of Zn homeostasis within the mealworms.

### Effect of Zn-feeding on larval Cu, Fe, Mn and Cd content

**Figure 3:**
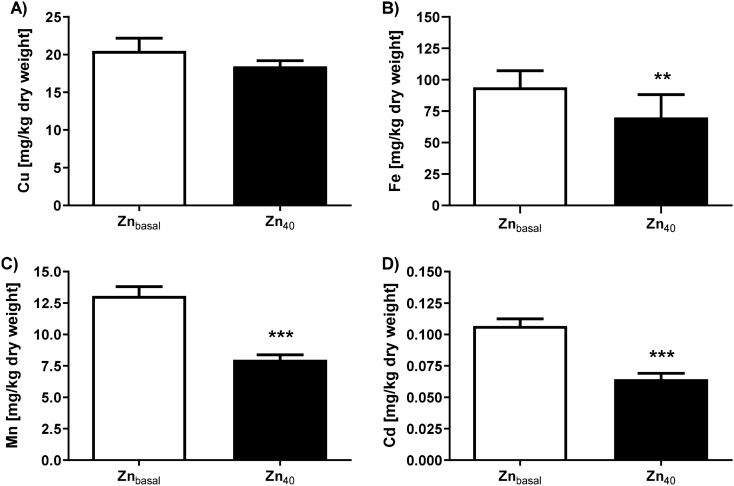
Concentrations of Cu, Fe, Mn and Cd in Zn-biofortified *T. molitor* larvae. *T. molitor* larval material obtained from the Zn_basal_ and Zn_40_ feeding groups was analyzed for (**A**) Cu, (**B**) Fe, (**C**), Mn and (**D**) Cd content. Data are shown as means + SEM of five replicates. Statistically significant differences from control are indicated (**p < 0.01, ***p < 0.001; Mann–Whitney test).

Increased Zn within the larval midgut might influence the uptake, transport and distribution of other metals. Feeding of the Zn_40_-spiked diet did not affect the copper status of the mealworm larvae (Fig. 3A; Zn_basal_ 20.5 ± 1.6 mg Cu kg^−1^ dry weight vs Zn_40_ 18.4 ± 0.7 mg Cu kg^−1^ dry weight). Fe content was 78.8 ± 8.4 mg kg^−1^ larval dry weight in the Zn_basal_ group. Administration of Zn_40_-wheat bran resulted in a decrease of larval Fe content to 59.1 ± 7.0 mg kg^−1^ larval dry weight (Fig. 3B; Mann–Whitney test: Fe [mg kg^−1^ dry weight] Zn_basal_ vs Zn_40_ p < 0.01). Likewise, Mn concentrations declined when providing Zn-enriched feed (Fig. 3C; Zn_basal_ 13.1 ± 0.7 mg Mn kg^−1^ dry weight vs Zn_40_ 8.0 ± 0.4 mg Mn kg^−1^ dry weight; Mann–Whitney test: Zn_basal_ vs Zn_40_ p < 0.001). Larval basal Cd content was 0.1 ± 0.0 mg kg^−1^ dry weight; almost the same as the wheat brain material (0.1 ± 0.0 mg Cd kg^−1^). Following feeding of the Zn_40_-wheat bran, 40% less Cd was detected within the L_9–10_ larval biomass (Fig. 3D; Mann–Whitney test: Cd [mg kg^−1^ dry weight] Zn_basal_ vs Zn_40_ p < 0.001). Lead was not detected within the larvae, neither in the Zn_basal_ nor in the Zn_40_ group.

## Discussion

The need to find innovative sources for food and feed production has led to an increased recognition of insects^2,27,28^. Currently, about 92% of the ~ 2000 edible insect species worldwide is wild harvested^29^. However, this is no longer an option with regard to economics of food and feed production, as there is a compelling need to farm the insects in mass quantities. Consequently, present-day insect industry is looking for reliable, consistent ways to scale their production in order to compete with other sources of livestock feed while guaranteeing safety and high nutritive quality of insects^2^.

*T. molitor* L. is a promising candidate and already grown by mass rearing^10,11^. There are several studies examining rearing conditions and the effect of different substrates on mealworm larval development^16,17,30,31^. The current study confirmed the larval fitness and growth response when grown on pure wheat bran^20^. The estimated FCR for the Zn_basal_ group (value 5.1) is almost identical to the one published by Melis et al. 2019 for wheat bran-fed mealworm larvae^32^, confirming the potency of this species to convert the bran feed into body weight of appropriate proximate composition^20,32^. However, dietary efficiency seems to be related to the micronutrient composition of the diet, as biomass output decreased with elevated Zn feeding. Feed avoidance due to excessive heavy metal concentrations (> 0.1% Zn^2+^) is common in terrestrial insects^21^, but was not observed in the present study. Recent observations depict the role of the Zn^2+^-gated midgut sensor Hodor in controlling *Drosophila melanogaster*s food intake behavior, to ensure insect growth even under macronutrient-scarce conditions^33^. This mechanism, however, seems to be of minor importance in the present study, as mealworms were fed with a rather high-calorie wheat bran diet.

Developmental retardation has already been observed in Zn feeding trials with other terrestrial insects^34,35^. This could be the outcome of systemic Zn intoxication. Besides, Zn might restrict the systemic availability of other essential minerals in the larvaes’ midgut^22,36^. Like any other organism, *T. molitor* requires trace elements for survival. A 6 ppm zinc supplementation was established by Fraenkel^37^ for optimal larval growth in synthetic casein/glucose-based minimal media. Hence, feeding wheat bran material containing a total of 89.4 mg Zn kg^−1^ (89.4 ppm) is seemingly an excess of Zn. However, the abundance of the anti-nutritive mineral antagonist phytate in wheat bran (estimated phytate/Zn molar ratio of 66) provides a rather unfavorable matrix for intestinal Zn absorption, decreasing its Zn bioavailability^38^. Still, the measured total Zn content for Zn_basal_ *T. molitor* larvae of around 120 mg kg^−1^ dry weight surpasses the wheat bran (Zn_larvae_/Zn_wheat bran_ ratio > 1). Accordingly, the mealworm larvae seem, at least partially, to abolish the Zn-antagonist throughout the digestion process, either by activating phytogenic phytase or phytases of gut-associated bacteria^17,39^. Feeding Zn-enriched wheat bran led to the intended Zn fortification of the insects, in the same order of magnitude as in a previous study^40^. Considering the estimated Zn-bioaccessibility upon human intestinal digestion of 40%^20^, an intake of ~ 30 g Zn_40_-freeze dried mealworms would be more than sufficient for an adult to replenish the daily zinc losses^41^. Due to their reduced total Fe content, consumption of Zn_40_-*T. molitor* larvae would result in lower Fe intake compared to food prepared from conventionally reared animals. Yet, the Fe content is still in excess of most other food sources, making even Zn_40_- larvae potentially useful for preventing Fe deficiency and contributing significantly to meeting the nutritional Fe requirements^42,43^. In any way, introducing Zn-biofortified mealworm larvae for use in food and feed will necessitate a copious assessment of the potential implications not just of their Fe content, but of their total macronutrient and vitamin composition as well as an examination of biological and chemical contaminants. This is crucial for avoiding long-term risks and adverse health effects for consumers^5–7^.

The Zn_larvae_/Zn_wheat bran_ ratio decreased inversely related to its concentration in the diet. Thus *T.molitor* larvae, similar to the mass rearing insect *Hermetia illucens*^44^, appear to be metal-deconcentrators^45^, able to adjust their internal Zn levels through homeostatic adaption of Zn absorption and excretion. Movement of Zn ions across the insect midgut epithelium, nowadays best described for the holometabolous insect *D. melanogaster*, requires the concerted interplay of solute carrier (SLC)39/Zrt, Irt-like protein (ZIP) Zn importers, SLC30/Zn transporter (ZnT) Zn exporters and cysteine-rich metal-binding metallothioneins (MT). Amongst the 10 ZIP and 7 ZnT proteins encoded in the *Drosophila* genome, the two importers dZIP42C.1 and dZIP42C.2, along with the exporter dZnT63C, are predominantly involved in dietary Zn absorption. Moreover, their expression is sensitive to both excessive and insufficient Zn supply^46^. Currently, 1219 insect genome-sequencing projects have been registered within the National Center for Biotechnology Information, among them 174 for *Coleoptera*, but the nuclear genome of *T. molitor* has not yet been unraveled^47^. According to our BLAST searches homologs of *D. melanogasters* dZIPs and dZnTs as well as the metal-responsive transcription factor-1 (dMTF-1) are encoded in *Tenebrionidae* genomes (Suppl Tables 1 and 2; Suppl Fig. 1–3). Notably, insects within this superfamily seem to lack the classical Cys-MTs, as the metal-buffering activity within midgut cells of *T. molitor* was shown to be mediated by other low molecular weight proteins rich in Asp/Glu^48^. Elucidating the expression, activity and tissue distribution of all these proteins during the lifecycle of *T. molitor* would not only contribute to the overall knowledge on Zn homeostasis in insects, but particularly provide decisive advances when aiming to raise the mealworm Zn enrichment quota by implementing Zn transporter-targeted strategies into rearing^49^.

Cu, Mn, and Fe contents of *T. molitor* larvae grown on non-spiked wheat bran were close to values reported for mealworm larvae in other feeding trials^19,50^, underlining the micronutrient quality of the applied bran material. Yet, the metals were differently impacted by Zn-biofortification. The Cu status of the mealworms remained stable, irrespective of Zn dosing. This is contrary to the situation in mammals, where prolonged high Zn feeding causes systemic copper-deficiency, possibly due to overexpression of intestinal apo-MT retaining the copper ions inside the enterocytes^51^. Insect midgut function seems to be strictly regionalized^52,53^. Cu uptake occurs primarily within the so-called “copper cell region”, where, at least in *D. melanogaster,* Zn was shown to slightly colocalize^33,54^. Nevertheless, the bivalent Zn^2+^ does not compete with cellular Cu uptake via the high-affinity Cu^+^ transporter Ctr1^55^. Cellular Zn-sensing by the metal regulatory transcription factor MTF-1 is critical with regard to triggering the expression of Zn-sensitive genes (e.g., metallothionein genes) in any species—including insects^56^. In addition to its conserved zinc fingers within the DNA-binding domain *D. melanogaster* MTF-1 contains a unique C-terminal cysteine-enriched copper-cluster that allows specific intracellular copper monitoring; consequently dMTF-1 keeps the cellular metallothionein gene transcription low under limited Cu supply and enhanced upon copper overload^57^. A homologous Cu-cluster sequence is present in the predicted protein of the beetle *Tribolium castaneum*, wheras *Asbolus verrucosus* MTF-1 is C-terminally truncated, thus missing this domain (Suppl Fig. 3). The outcome of the 1KITE (1 K Insect Transcriptome Evolution) and the “i5k” (Sequencing Five Thousand Arthropod Genomes)^58^ initiative will soon provide insight into the regulation of Zn and Cu homeostasis in several other insects designated for food/feed production.

The reduction in total Fe and Mn content in the Zn_40_ larvae is probably due to a competition with Zn for intestinal transporters at the site of absorption. Malvolio (Mvl), an insect homolog of the divalent metal ion transporter 1 (DMT1), is the likeliest candidate for the luminal uptake of Fe^2+^and Mn^2+^ ^59^ in the mealworm midgut (see Suppl Table 2). Although Zn^2+^ is a rather weak Mvl substrate^60^, there might be a competition with the aforementioned metal ions, especially under massive nutritional Zn excess. Accordingly, a Zn/Fe/Mn-mixed co-supplementation strategy during mealworm rearing would be a worthwhile option when aiming to counterbalance the other trace element delivery quotas while simultaneously enhancing larval growth/biomass output.

As *T. molitor* larvae are aimed to be rated as novel food or feed, further quality and safety concerns should be addressed and strictly monitored in addition to their macro- and micronutrient content. In fact, insects might accumulate hazardous chemicals during growth, amongst them heavy metals that may pose a risk to humans or animals^7,61^. As a natural resource, grain material varies considerably in its elemental composition, which is also reflected in slightly different toxic metal contents in *T. molitor* within various studies^61^. The EU maximum level for Cd in complete feed for farm animals has been set at 0.5 mg kg^−1^ (relative to a moisture content of 12%)^62^. From a safety perspective, the mealworm larvae grown on Zn_basal_ wheat bran in the present study would be acceptable for feed application, consistent with results of other studies using the same feeding substrate^63,64^. Insects intended for human consumption in Europe are covered by the Novel Food Regulation EU 2015/2283^3^, and must therefore be authorized by EU institutions^61^. The EU “ALARA" (as low as reasonably achievable policy) precautionary principle becomes relevant when setting maximum limits for contaminants, including heavy metals, in foodstuff for public health protection purposes (Regulation 315/93/EEC^65^). The introduction of Zn-enriched wheat bran material into mealworm breeding is certainly an effective strategy to reduce the amount of the human/animal toxicant Cd within the mealworm larvae. Zn-competitive transport routes for cellular Cd uptake in insects were already discussed^66^, yet their molecular identity and mechanisms need to be clarified when intending to further target the Cd burden of insects during rearing. Aside from interfering with the uptake of this toxic element, Zn triggers synthesis of the aforementioned MT-like proteins within the midgut epithelium, providing a stable pool of midgut Cd-trapping molecules. In fact, these proteins were described to be released into the feces during cell gut renewal^44,48,63^, explaining the decreased mealworm larval Cd bioaccumulation factor observed in the present study. As a substantial part of Cd enters the cells via Ca^2+^ channels^67^, it might be useful to include this macromineral in future mealworm enrichment strategies. Here, Zn/Fe/Cu/Mn/Ca-supra-supplemented wheat bran should provide an ideal trace element composition when aiming to produce mealworm larvae enriched in multiple essential micronutrients and low in toxic metals, which is worthwhile to be tested and introduced into *T. molitor* rearing on an industrial scale.

## Conclusion

Global population growth will increasingly challenge the food industry in the coming years. The yellow mealworm (*T. molitor*) is a sustainable alternative source to animal-derived protein and lipids, suitable for mass rearing and large-scale industrial production. Yet, the industrial technology needs to be optimized and standardized to process the insects in the best possible way, from an economic, food/feed safety and nutritive point of view. Summarizing this study’s results, a Zn-spiked wheat bran feeding strategy is an easy and inexpensive approach to produce *T. molitor* valorized in its content of the essential micronutrient Zn. Zn-biofortification led to a moderate Fe and Mn reduction in mealworms, reflected in reduced feed conversion efficiency. Nevertheless this can easily be counteracted in the future by Fe/Mn co-supplementation. Importantly, Zn-enriched rearing reduced the levels of the human/animal toxicant Cd within the mealworm larvae, a technological novelty of outstanding importance to be implemented in the future production process to ensure the consumer safety of this edible insect species. Overall, these results provide relevant insight for the development of optimized strategies to process *T. molitor* in the future, both in terms of nutrient quality and quantity.

## Materials and methods

### Materials and chemicals

Wheat bran/wheat flour (Roland Mills Nord GmbH & Co. KG, Bremen, Germany); H_2_O_2_ (Sigma Aldrich, Munich, Germany); HNO_3_ (Sigma Aldrich, Munich, Germany); Indium Standard for ICP-MS TraceCERT (Sigma Aldrich, Munich, Germany); Multielement Standard Solution 6 for ICP TraceCERT (Sigma Aldrich, Munich, Germany); Rhodium ICP-MS Standard TraceCERT (Sigma Aldrich, Munich, Germany); ZnSO_4_∙7H_2_O (Sigma Aldrich, Munich, Germany).

### Experimental design

*T. molitor* beetles of University of Applied Sciences Bremerhaven own breeding were placed on wheat flour for oviposition (see Fig. 1). After 7 days, the eggs were removed from the laying substrate with a fine-mesh sieve (1 mm mesh size) and placed in the breeding container. Freshly hatched larvae were allowed to grow in the wheat bran substrate. 6 weeks post hatching *T. molitor* larvae (stage L_3–4_, average starting weight 1.23 ± 0.01 mg) were seeded into 400 ml glass beakers on Zn-spiked wheat bran feed (Zn_basal_ to Zn_40fold basal_; further details for wheat bran preparation are provided in the “feed spiking” section). Each Zn treatment was performed in 5 biological replicates, where each replicate comprised 60 mealworm larvae in a density of 142 larvae/dm^2^ growth surface. All beakers were incubated at 27 °C with a humidity of 75% with no day/night rhythm for light, temperature and humidity for an 8 week period^68^. Each feeding group was fed as soon as the feed in the beaker had been consumed, with a total of 20 g feed over the whole feeding period. The exact amount of feed supply and feces production was recorded separately for every larval replicate. After harvesting at day 98 post hatching, the number and larval weight of living L_9–10_ animals was registered to assess the growth performance and feed utilization. Prior to metal analysis, larvae were starved for 24 h to diminish gastrointestinal feed residues. Following freeze-drying in a Christ Beta 1–8 LD Plus freeze dryer (Martin Christ, Osterode am Harz, Germany)^20^ all samples were stored at − 20 °C.

### Feed spiking

Wheat bran already contains micronutrients, including Zn. Thus the basal Zn content of this study’s wheat bran batch was evaluated prior to spiking. To this end, 500 mg wheat bran samples were subjected to a microwave-assisted digestion (Mars 6, CEM GmbH, Kamp-Lintfort, Germany) with a 1:1 mixture of ultrapure HNO_3_ (65%) and H_2_O_2_ (30%). Zn content was analyzed by flame atomic absorption spectrometry (FAAS) on a Perkin Elmer AAnalyst 800 (Perkin Elmer, Rodgau, Germany) applying an external calibration (analytical parameters: LOD 10.3 µg Zn/l; LOQ 15.9 µg Zn/l;^20^). Based on these values, spiking of 500 g wheat bran samples with ZnSO_4_·7H_2_O pre-solved in 50 ml 18.2 MΩ·cm water (Millipore Milli-Q Water Purification System) was then performed to reach the desired final Zn biomass concentration ranging between 89.4 mg/kg wheat bran (Zn_basal_) to a maximum of 3577.5 mg/kg wheat bran (Zn_40fold basal_) (see Table 2).

**Table 2:** Zn concentrations in the non-spiked and Zn-enriched wheat bran.

Treatment	Zn_basal_	Zn_2.5_	Zn_5_	Zn_7.5_	Zn_10_	Zn_15_	Zn_20_	Zn_25_	Zn_30_	Zn_40_
ZnSO_4*_7H_2_O spike [mg/kg]	–	590.0	1573.5	2556.9	3540.3	5507.2	7474.0	9440.9	11,407.7	15,341.4
Zn^2+^ [mg/kg]	89.4	223.6	447.2	670.8	894.4	1341.6	1788.7	2235.9	2683.1	3577.5

### Larval growth performance and feed utilization parameters

Larval growth performance was evaluated based on the larval fresh weight and the survival rate (SR, Eq. 1) of L_9–10_ animals.

1 $$SR = \frac{{number\,of\,surviving{ }L_{9 - 10} { }larvae}}{{number\,of\,L_{3 - 4} { }larvae\,seeded}} \times 100{\text{\% }}$$

Larval weight data from the end (stage L_9–10_) and the beginning (stage L_3–4_) of the feeding period along with total feed consumption (FC, Eq. 2) were further used to calculate the efficiency of ingested feed conversion (ECI, Eq. 3) and the feed conversion ratio (FCR; feed input per unit of fresh product; Eq. 4) on fresh matter basis^16,31,69^.

2 $$FC\left[g\right]={total\,weight\, feed}_{provided} \left[g\right]- {total \,weight\, feed}_{unconsumed} \left[g\right]$$

3 $$ECI=\frac{{{weight}_{L9-10 larvae }\left[g\right]- weight}_{L3-4 larvae}\left[g\right]}{FC \left[g\right]}\times 100\%$$

4 $$FCR=\frac{total \, {feed}_{ingested} \left[g\right]}{{{weight}_{L9-10 larvae }\left[g\right]- weight}_{L3-4 larvae}\left[g\right]}$$

Feed assimilation rate (FA, Eq. 5) was used to evaluate the efficiency of digested feed conversion (ECD; Eq. 6;^16,69^)

5 $$FA\left[g\right]=FC \left[g\right]-{total\,weight}_{faeces} \left[g\right]$$

6 $$ECD=\frac{{{weight}_{L9-10 larvae }\left[g\right]- weight}_{L3-4 larvae}\left[g\right]}{FA \left[g\right]}\times 100\%$$

### Trace element analyses of mealworm larvae

Larval materials were grinded replica-wise and portioned before heating in a laboratory microwave digester (Mars 6, CEM GmbH, Kamp-Lintfort, Germany) in a 3:1 mixture of ultrapure HNO_3_ (65%) and H_2_O_2_ (30%) containing 500 µg/L indium as an internal standard to estimate metal recovery rates. After digestion the samples were prediluted to 5 ml using 18.2 MΩ·cm water (Millipore Milli-Q Water Purification System). For Zn, Cu, Mn and Cd quantification samples were further diluted 1:100 in 0.65% HNO_3_ containing 5 µg/L rhodium and analyzed on an Elan DRC II inductively coupled plasma-mass spectrometer (PerkinElmer LAS GmbH, Rodgau, Germany). For Fe quantification, 1:500 prediluted samples were analyzed in DRC mode using methane as reaction gas. Further ICP-MS conditions are listed in Table 3. The instrument was tuned daily for maximum sensitivity (< 0.03 oxide ratio (^140^Ce^+ 16^O/^140^Ce^+^), double charged ratio < 0.03 (^137^Ba^++^/^137^Ba^+^) and background counts < 2 cps).

**Table 3:** Experimental conditions for ICP-MS measurements.

Forward power	1550 W
Cool gas flow	15 L min^−1^
Auxiliary gas flow	0.9 L min^−1^ (Argon)
Nebulizer gas flow	0.9 L min^−1^ (Argon)
Nebulizer type	MicroMist
Quadrupole (m/z)	66 (Zn); 57 (Fe); 55 (Mn); 63 (Cu); 111 (Cd); 103 (Rh); 115 (In)
DRC gas flow	Methane, 1 L min^−1^ (0.75 Rpq)
Limit of quantitation	0.2 µg L^−1^ (Zn); 2 µg L^−1^ (Fe); 0.1 µg L^−1^(Mn); 0.5 µg L^−1^ (Cu); 0.15 µg L^−1^ (Cd)
Calibration range	1–100 µg L^−1^ (Zn, Fe, Mn, Cu); 0.01–1 µg L^−1^ (Cd)

### Statistical analyses

Statistical significance of the experimental results was analyzed by either Mann–Whitney U test or one-way ANOVA/Dunnett’s post hoc test using GraphPad prism software version 8.02 (GraphPad Software Inc., CA, USA).

## Supplementary information

Supplementary Information
