# Supplementary material for: Dietary zinc enrichment reduces the cadmium burden of mealworm beetle (Tenebrio molitor) larvae
Source: Sci Rep. 2020 Nov 18;10:20033. doi: 10.1038/s41598-020-77079-x (PMC7674442; doi:10.1038/s41598-020-77079-x)
Supplement: Supplementary file 1 — Supplementary Information [file 41598_2020_77079_MOESM1_ESM.docx]

**Dietary zinc enrichment reduces the cadmium burden of mealworm beetle (*Tenebrio molitor*) larvae**

**Supplemental material**

Claudia Keil^1#^, Maria Maares^1#^, Nina Kröncke^2^, Rainer Benning^2^, Hajo Haase^1*^

^1^Technische Universität Berlin, Institute for Food Technology and Food Chemistry, Berlin, Germany

^2^ University of Applied Sciences Bremerhaven, Institute of Food Technology and Bioprocess Engineering, An der Karlstadt 8, 27568 Bremerhaven, Germany

^# Both authors contributed equally to this paper^

*Corresponding author: haase@tu-berlin.de; Tel.: +49 (0) 30 31472788; Fax: +49 (0) 30 31472823

Sequences of *Drosophila melanogaster* proteins involved in Zn homeostasis (Suppl Table 1; for nomenclature see Xiao et al. 2016 ^1^ and Navarro & Schneuwly 2017 ^2^) were annotated into a series of BLAST searches (<https://blast.ncbi.nlm.nih.gov/Blast.cgi>) to identify potential homologues encoded in currently listed genomes of Tenebrionidae (NCBI data base (<https://www.ncbi.nlm.nih.gov/> ^3^) (see Suppl Table 2). The prediction of transmembrane domains was done using the Phobius Web Server (<https://www.ebi.ac.uk/Tools/pfa/phobius/> ^4^). The PROSITE database (<https://prosite.expasy.org/> ^5^) was used to predict Zn finger motives. Selected amino acid sequences were aligned using the Clustal omega Multiple Alignment tool (<https://www.ebi.ac.uk/Tools/msa/clustalo/>; ^6^). Phylogenetic analyses were performed with the program NGPhylogeny.fr (<https://ngphylogeny.fr/> ^7^)

**Suppl Table 1: Drosophila melanogaster proteins involved in Zn homeostasis**

| **Solute carrier (SLC)39/Zrt Irt-like protein (ZIP) Zn importers** | | | | |
| --- | --- | --- | --- | --- |
| **dZip42C.1**  NP_525107.1 | **dZip42C.2**  NP_610231.2 | **dZip89B**  NP_536747.2 | **dZip88E**  NP_650440.2 | **dZIP71B**  NP_001303364.1 |
|  |  |  |  |  |
| **foi**  NP_001286985 | **catsup**  NP_524931 | **dZIP99C**  NP_733323 | **dZIP102B**  NP_001284713.1 | **dZIP48C**  NP_610712.1 |
|  |  |  |  |  |
| **SLC30/Zn transporter (ZnT) Zn exporters** | | | | |
| **dZNT63C**  NP_728856.1 | **dZnT77C**  NP_649233.2 | **dZnT33D**  NP_723732.2 | **dZnT35C**  NP_609741.3 | **dZnT41F**  NP_724429.1 |
| **dZnT86D**  NP_650049.1 | **dZnT49B**  NP_725207.1 |  |  |  |
| **Metallothioneins** | | | | |
| **MtnA**  NP_524299.1 | **MtnB**  NP_524413.1 | **MtnC**  NP_650882.1 | **MtnD**  NP_788695.2 | **MtnE**  NP_001189254 |
| **metal-responsive transcription factor-1 (MTF-1)** | | | | |
| **dMTF-1**  NP_001097560.1 |  |  |  |  |
| **Malvolio** | | | | |
| **dMvl**  NP_001163668.1 |  |  |  |  |

**Suppl Table 2: Protein candidates potentially involved in regulation of Tenebrionidae Zn homeostasis**

| **Protein**  **Family** | **NCBI accession** | **Gene ID^a^** | **Chromosome** | **Protein length** | **characteristics** |
| --- | --- | --- | --- | --- | --- |
| ***Tribolium castaneum*** | | | | | |
| **SLC39/ZIP Zn importers** | XP_008190957.1 | LOC663579 | 3 | 330 | 8 TM domains |
|  | XP_974693.1 | LOC663561 | 3 | 325 | 8 TM domains |
|  | KYB28661.1 | LOC655157 | 3 | 314 | 7 TM domains |
|  | XP_974675.1 | LOC663543 | 3 | 384 | 8 TM domains |
|  | XP_969488.1 | LOC657975 | 7 | 373 | 8 TM domains |
|  | XP_008191263.1 | LOC659638 | 3 | 343 | 8 TM domains |
|  | XP_008195606.1 | LOC659472 | 7 | 608 | 6 TM domains |
|  | XP_974198.1 | LOC663042 | 5 | 339 | 6 TM domains |
|  | XP_975125.2 | LOC664008 | 9 | 315 | 7 TM domains |
|  | XP_972841.1 | LOC661597 | 6 | 282 | 8 TM domains |
| **SLC30/ZnT Zn exporters** | XP_970510.2 | LOC659085 | 4 | 421 | 6 TM domains |
|  | XP_008192604.1 | LOC662439 | 4 | 464 | 6 TM domains |
|  | XP_015839451.1 | LOC655654 | X | 416 | 6 TM domains |
|  | EFA06430.1 | LOC663791 | 7 | 382 | 6 TM domains |
|  | XP_968324.1 | LOC656722 | 7 | 346 | 6 TM domains |
|  | XP_975475.2 | LOC664375 | 4 | 619 | 4 TM domains |
| **MTF-1** | XP_008193541.1 | LOC103313055 | 5 | 646 | 6 C2H2-type Zn fingers |
| **Malvolio** | XP_967521.1 | LOC655866 | 6 | 564 | 12 TM domains |
| ***Asbolus verrucosus*** | | | | | |
| **SLC39/ZIP Zn importers** | RZC37672.1 |  |  | 264 | 6 TM domains |
|  | RZC38782.1 |  |  | 335 | 8 TM domains |
|  | RZC40892.1 |  |  | 288 | 6 TM domains |
|  | RZC37372.1 |  |  | 368 | 8 TM domains |
|  | RZC34723.1 |  |  | 342 | 6 TM domains |
|  | RZC43028.1 |  |  | 361 | 3 TM domains |
|  | RZC38724.1 |  |  | 211 | 4 TM domains |
|  | RZC42734.1 |  |  | 216 | 6 TM domains |
|  | RZC37794.1 |  |  | 232 | 3 TM domains |
| **SLC30/ZnT Zn exporters** | RZC33991.1 |  |  | 447 | 6 TM domains |
|  | RZB41618.1 |  |  | 287 | 4 TM domains |
|  | RZC37549.1 |  |  | 390 | 6 TM domains |
|  | RZB89864.1 |  |  | 369 | 4 TM domains |
|  | RZB39981.1 |  |  | 350 | 6 TM domains |
|  | RZC38739.1 |  |  | 553 | 4 TM domains |
| **MTF-1** | RZC38605.1 |  |  | 478 | 8 C2H2-type Zn fingers |
| **Malvolio** | RZC14257 |  |  | 543 | 12 TM domains |


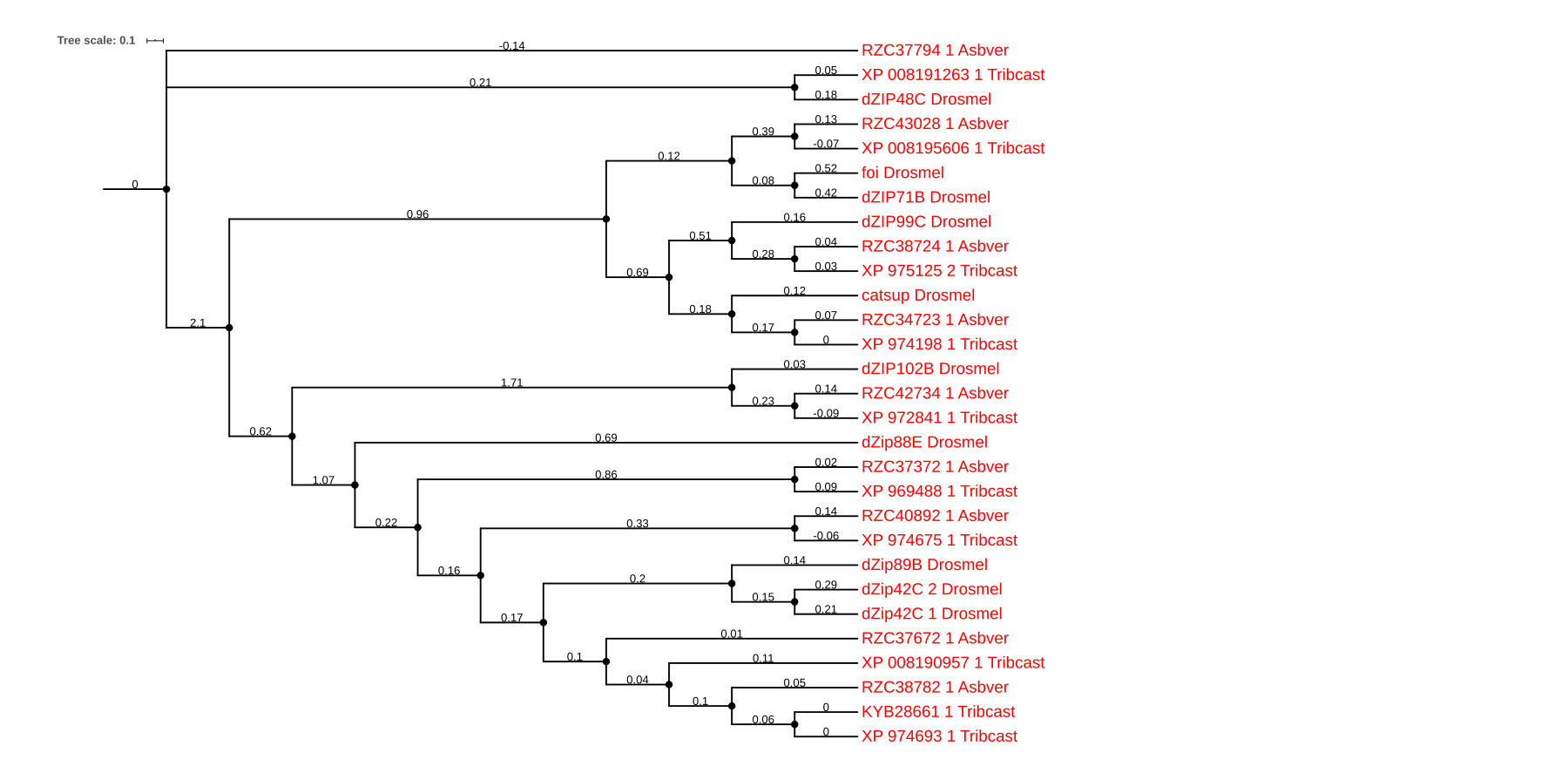


**Suppl. Fig. 1: Phylogenetic tree revealing the relationship between *Drosophila melanogaster* ZIP family members and Tenebrionidae protein candidates**

Drosmel= *Drosophila melanogaster*; Asbver= *Asbolus verrucosus*, Tribcast= *Tribolium castaneum;* see the previous reports from Lye et al. 2012 ^8^ and Qin et al. 2013 ^9^ for phylogenetic tree analysis of *Drosophila melanogaster* ZIPs.


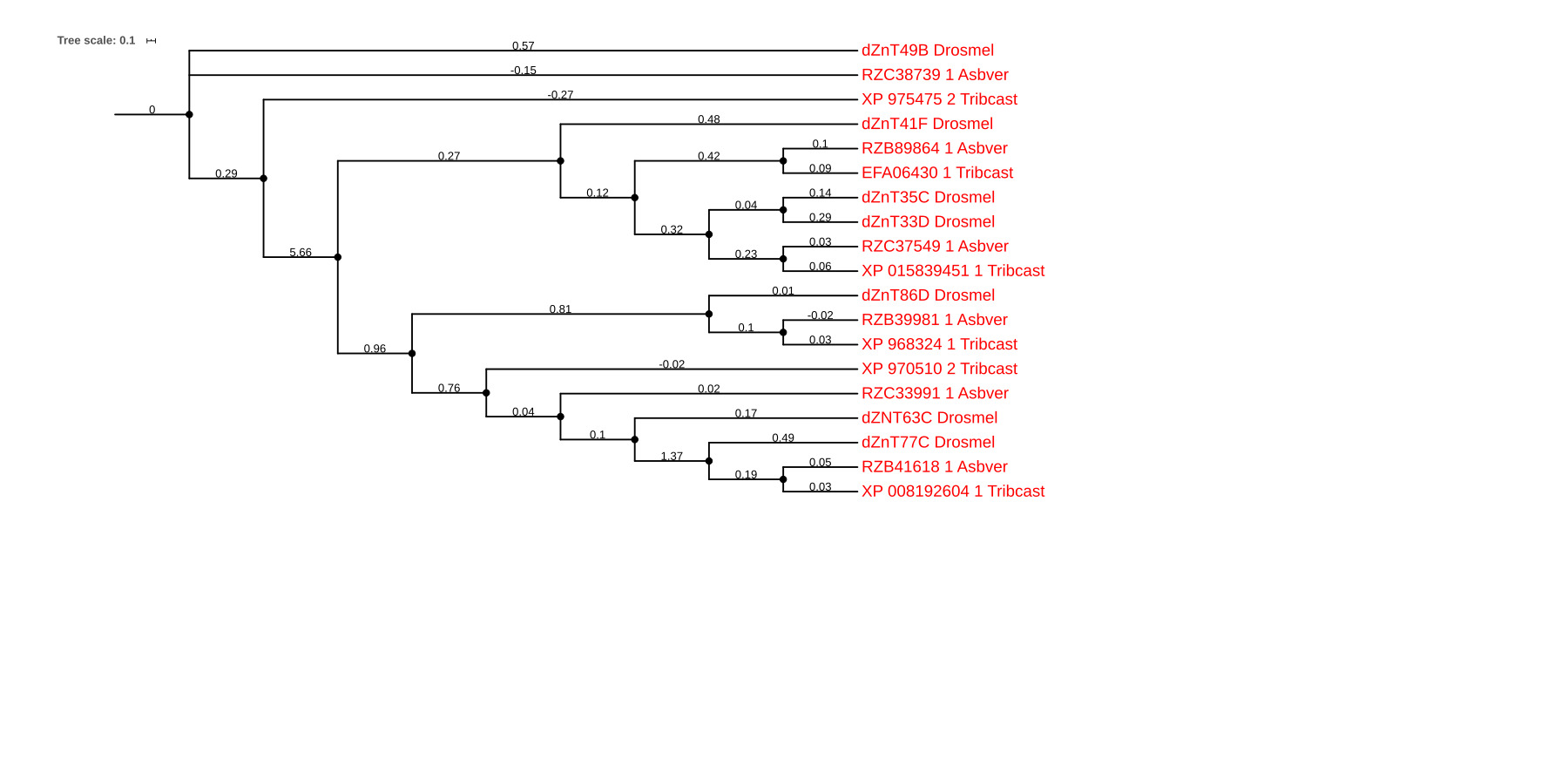


**Suppl. Fig. 2: Phylogenetic tree revealing the relationship between *Drosophila melanogaster* ZnT family members and Tenebrionidae protein candidates**

Drosmel= *Drosophila melanogaster*; Asbver= *Asbolus verrucosus*, Tribcast= *Tribolium castaneum;* see the previous reports from Lye et al. 2012 ^8^ and Qin et al. 2013 ^9^ for phylogenetic tree analysis of *Drosophila melanogaster* ZnTs.

Drosmel MNDQEKQHQQQQQHYFHSEWKTTANSHGNSLSDSRDYDTNSSRNSLSGSPPVTSNCSSSG 60

Tribcast ------------------------------------------------------------ 0

Asbver ------------------------------------------------------------ 0

Drosmel SLDFDRPLAQLLEPKLEADGLGIIHGDNYSIYGSQTAESTSGEQQVLNLGLTLDTGDAQA 120

Tribcast ------------------------------------------------------------ 0

Asbver ------------------------------------------------------------ 0

Drosmel TYGDLFGAQDQLTASQTHGHSQVVVSAAADNAFSLADQLTPLPITVIPITYHHTTENLSS 180

Tribcast ------------------------------------------------------------ 0

Asbver ------------------------------------------------------------ 0

Drosmel SHSEVPLIASLSDITAQVFNLDDICFTLEYQFENQRLVQVQPPTVVSLSMVSSPNEAANP 240

Tribcast ------------------------------------------------------------ 0

Asbver -----------------------------------------MP-----EMVGYYNEATLP 14

Drosmel PINCDNDQGTGNSISRDSTSNSPAYFTIETSYVDEYDPNEPDEDEQLAHCIQPGVLHQDV 300

Tribcast ------------------------------------------------MHIHPGASKKMP 12

Asbver IATVQTADEA--RLPVKKV------VCSPDLPIAEYPPGGNRQPEQIV-NIQ**C**QI**C**NKMY 65

*: ::

**ZF1**

Drosmel DEEEVERQDENDQLMALAYESSDEALSRYR**C**NYEN**C**YRSYSTIGNLRT**H**LKT**H**TGDYSFK 360

Tribcast EKP----SHATLTITSTDPTTKETTVNRYN**C**EYDG**C**TRTYSTVGNLRT**H**MKT**H**KGEYRFK 68

Asbver NSK--------AAFQA**H**QRT**H**AKETDDPYR**C**N--I**C**SKTFAVPARLTR**H**YRT**H**TGEKPFR 115

:. : : . : . *.*: * ::::. ..* * :**.*: *:

**ZF2 ZF3**

Drosmel **C**PEDG**C**HKAFLTSYSLKI**H**VRV**H**TKVKPYE**C**EVSG**C**DKAFNTRYRLHA**H**LRL**H**NGETFN**C** 420

Tribcast **C**TESN**C**GKAFLTSYSLKI**H**IRV**H**TKVKPFE**C**KSPG**C**DKAFNTLYRLRA**H**ERL**H**NGKTFN**C** 128

Asbver **C**E--F**C**NKSFSVKENLSV**H**RRI**H**TKERPYK**C**EV--**C**SRAFEHSGKLHR**H**MRI**H**TGERPH- 170

* * *:* .. .*.:* *:*** :*::*: *.:**: :*: * *:*.*: :

**ZF4 ZF5**

Drosmel E--L**C**QKCFTTLSDLKK**H**MRT**H**TQERPYK**C**PEDD**C**GKAFTASHHLKT**H**RRT**H**TGEKPYP**C** 478

Tribcast ESEG**C**MKFFTTLCDLKK**H**TRT**H**TREKPYK**C**KEDG**C**GKAFTASHHLKT**H**QRI**H**SGEKPYI**C** 188

Asbver K**C**TI**C**SKTFIQSGQLVI**H**MRT**H**TGEKPYV**C**T--V**C**QKGFTCSKQLKV**H**SRT**H**TGEKPYS**C** 228

: * * * :* * **** *:** * * *.**.*::**.* * *:***** *

**ZF6**

Drosmel QEDS**C**QKSFSTS**H**SLKS**H**KKTHQRQLQNK-GRKKRPLK---TQQTKCSDQEQKDPQQEEQ 534

Tribcast KESS**C**SRAFATL**H**SLKS**H**IKTHQKSELKLEGQEEKDD----SVKPELGENERRNN----D 240

Asbver E--I**C**GKSFGYN**H**VLKL**H**QVAHYGEKVYK**C**TI**C**NDTFNSKKSMEA**H**IKS**H**---------S 277

: * ::*. * ** * :* . : : : . .: .

Drosmel EEEEFIKEDQPEMTLLNPGSHC------SETTSTDSGVVLQT-----------LTP---Q 574

Tribcast INVPFDFEGS--YTIGSFGSALDTWDDLEKAESKKSDVTTKQVPEPNNLTESNLEPVNFD 298

Asbver ENAPTA--PTPPASSTSSESSCSSSS--SDKENK------DSLPLP-------QEPTSYD 320

: : . * .. .. . * :

Drosmel DHLSN-------VFILQGNEPLILPETSQAYQLSYA--A--EEEIPS-PWIDAGVLVSKP 622

Tribcast NLFKNSYNLVNNNYIARNPD-ASLPPLTLDNKAKYA--AVIEADLSS-QFEMANGLKNY- 353

Asbver SDIRY--Y----IYPRDRLSPVYVPQSYAGAGVELLATAATEDKFRSAPDVVFNIITKNP 374

. : : . :* . * * .: * . : .

Drosmel IIPMAPLTDACVALPTEMPSFVNLKPTFGNAVSGNMGDPQPE---------TMDVD-PTV 672

Tribcast ----A-TVNTAEPIPTQLPYNIGTE----NIENGKAGETLND--------TQMELEDSSI 396

Asbver QEVMTAL-----RH---PAYFTAPTVQYTPL---NAGDDIRKKVEAVLAADEIQSEEENI 423

: : *: . :: : .:

Drosmel ET----------SLPTPTLELNQPNIEELLKQD--------------------------S 696

Tribcast IT----------EIEDAGINFDVDMFDNVFNDDKNKKVNVISVKKIVPPENDLVNLDKQI 446

Asbver LTPPSSNPVSPAPSPDPELSLPPRKRSKMILK---------------------------- 455

* :.: .::: .

Drosmel FEN-------EEDME-TESLLNDILMT-------------IDNNSALL-QATLQQAS--- 731

Tribcast YTPEALQMSLACDEE-VPSMWVDVMNYYNSGQANVFEQNTIDDSQVIAVPTAVQSYVNLP 505

Asbver --------SMEATIDLSPVRYSSVIQYAGAS----------------------------- 478

: .::

**Tetranuclear**

**Cu(I)cluster**

Drosmel -------QVPS-------DASGLVELDIRDNKPTLKQITADAGI**C**N**C**TN**C**K**C**DQTKS**C**HG 777

Tribcast PLQTTDNQLDSTYNQLNSFLMQNLEQTTNTDANLLKNLTAEADI**C**K**C**VD**C**K**C**DSVNN**C**QN 565

Asbver ------------------------------------------------------------ 478

Drosmel GD**C**GAAA---AQQTTRTGAPTTTTTTTTMNSSGGKRICGSVVPTKKVSKRETEMNQNIED 834

Tribcast **C**EGHDRAEAPQAQNCQSKVSCCSKPVKNLGCGGDKNDCCVVVCLKTLDQLRQ-------- 617

Asbver ------------------------------------------------------------ 478

Drosmel VALLLQNLASMSSGGSSGGGGGCCGGGAVKPAPSGGGCCGEPKAPKPVNAGCGCARPSAP 894

Tribcast ----ILNMAS-TCGGFQNLTLGCIKGGQF---------CAVQK----------------- 646

Asbver ------------------------------------------------------------ 478

Drosmel SSGGCCGSGVASTPKPTGGCCSGNQKPAPTATAASSPSCCCSGKGEVQEPPVISSASSAP 954

Tribcast ------------------------------------------------------------ 646

Asbver ------------------------------------------------------------ 478

Drosmel AAAVKGNACTCKSPAEGVANGCCVVICIKTLQALRKVLTRRNLNLMLCPQQN 1006

Tribcast ---------------------------------------------------- 646

Asbver ---------------------------------------------------- 478

**Suppl. Fig. 3: Sequence alignment of the *Drosophila melanogaster* MTF-1 and Tenebrionidae protein candidates**

Drosmel= *Drosophila melanogaster* (accession number NP_001097560.1); Asbver= *Asbolus verrucosus* (accession number RZC38605.1), Tribcast= *Tribolium castaneum* (accession number XP_008193541.1)*;* The nomenclature of the MTF-1 Zn fingers for the drosophila protein is taken from Zhang et al. 2001 ^10^. The Cys-rich Cu(I) domain is described in Chen et al. 2008 ^11^.

**References**

1. Xiao, G. & Zhou, B. What can flies tell us about zinc homeostasis? *Arch. Biochem. Biophys.* **611**, 134–141 (2016).

2. Navarro, J. A. & Schneuwly, S. Copper and zinc homeostasis: Lessons from Drosophila melanogaster. *Front. Genet.* **8**, 1–20 (2017).

3. National Center for Biotechnology Information (NCBI). Available at: https://www.ncbi.nlm.nih.gov/genome/browse/#!/overview/.

4. Käll, L., Krogh, A. & Sonnhammer, E. L. L. Advantages of combined transmembrane topology and signal peptide prediction-the Phobius web server. *Nucleic Acids Res.* **35**, 429–432 (2007).

5. A Sigrist, C. J. *et al.* New and continuing developments at PROSITE. *Nucleic Acids Res* **41**, D344-7 (2013).

6. Sievers, F. & Higgins, D. G. Clustal omega, accurate alignment of very large numbers of sequences. *Methods Mol. Biol.* **1079**, 105–116 (2014).

7. Lemoine, F. *et al.* NGPhylogeny.fr: new generation phylogenetic services for non-specialists. *Web Serv. issue Publ. online* **47**, W260–W265 (2019).

8. Lye, J. C. *et al.* Systematic functional characterization of putative zinc transport genes and identification of zinc toxicosis phenotypes in Drosophila melanogaster. *J. Exp. Biol.* **215**, 3254–3265 (2012).

9. Qin, Q., Wang, X. & Zhou, B. Functional studies of Drosophila zinc transporters reveal the mechanism for dietary zinc absorption and regulation. *BMC Biol.* **11**, 101 (2013).

10. Zhang, B. O., Egli, D., Georgiev, O. & Schaffner, W. The Drosophila Homolog of Mammalian Zinc Finger Factor MTF-1 Activates Transcription in Response to Heavy Metals. *Mol. Cell. Biol.* **21**, 4505–4514 (2001).

11. Chen, X. *et al.* Copper sensing function of Drosophila metal-responsive transcription factor-1 is mediated by a tetranuclear Cu(I) cluster. *Nucleic Acids Res.* **36**, 3128–3138 (2008).
